# PREDator - a new structure-based approach for cross-reactivity predictions

**DOI:** 10.1186/1758-2946-5-S1-P6

**Published:** 2013-03-22

**Authors:** Florian Koelling

**Affiliations:** 1 Institut für Medizinische und Pharmazeutische Chemie, Technische Universität Braunschweig, Beethovenstr. 55, 38106 Braunschweig, Germany

## 

Ligandcentric virtual screening techniques employ in most cases three-dimensional Gaussians in order to define a molecule's entire pharmacophoric properties (ideally of a co-crystallised ligand) [1] and have been applied successfully in many prospective and retrospective drug discovery campaign [2].

Here, the development of a new pharmacophoric binding site descriptor in the spirit of Cavbase [3] is presented: Instead of focussing on ligand features, crucial amino-acid residues within the binding site are identified and represented as a pharmacophore model. Our method aims to combine the advantages of Cavbase with the smooth nature of a Gaussian pharmacophore representation, thus enabling binding site comparisons independently of sequence homology. Gaussian models are fast to compute and show the advantage that only very few parameters have to be defined. In contrast to a recently published approach where the entire binding site is defined by a Gaussian model for structure-based cross-reactivity predictions [4], PREDator employs only a few characteristic cavity-flanking amino acids [5] which are finally encoded in order to accelerate computations.

It is shown that these models, as a conceptual representation of the binding site, can be used successfully for cross-reactivity predictions. Compared to a ligandcentric approach [5] with regard to this purpose, a structure-based approach is advantageous in terms of being less dependent on the ligand-scaffold.
